# Near infrared indocyanine green fluorescent cholangiography versus intraoperative cholangiography to improve safety in laparoscopic cholecystectomy for gallstone disease—a systematic review protocol

**DOI:** 10.1186/s13643-022-01907-6

**Published:** 2022-03-03

**Authors:** Mihai-Calin Pavel, Mar Achalandabaso Boira, Yasir Bashir, Robert Memba, Erik Llácer, Laia Estalella, Elisabeth Julià, Kevin C. Conlon, Rosa Jorba

**Affiliations:** 1 Hepato-Pancreato-Biliary Unit, General and Digestive Surgery Department, University Hospital of Tarragona Joan XXIII, C/ Dr. Mallafrè Guasch, 4, 43005 Tarragona, Spain; 2 Departament of Medicine and Surgery, Universitat Rovira i Virgili, Reus, Spain; 3 Department of Surgery, University Hospital Marques de Valdecilla, University Cantabria, Santander, Santander, Spain; 4 Department of Surgery, Trinity College Dublin, Tallaght Hospital, Dublin, Ireland; 5 School of Technical Engineering, Universitat Rovira i Virgili, Reus, Spain

**Keywords:** Indocyanine Green, ICG, Fluorescence, Cholecystectomy, Bile duct injury

## Abstract

**Background:**

Laparoscopic cholecystectomy has become the standard surgical approach in the treatment of cholelithiasis. Diverse surgical techniques and different imaging modalities have been described to evaluate the biliary anatomy and prevent or early detect bile duct injuries. X-ray intraoperative cholangiography (IOC) and near infrared indocyanine green fluorescent cholangiography (NIR-ICG) are safe and feasible techniques to assess biliary anatomy. The aim of this systematic review will be to evaluate if NIR-ICG can visualize extrahepatic biliary anatomy more efficiently and safer than IOC in minimally invasive cholecystectomy for gallstone disease.

**Methods:**

Literature search will be performed via MEDLINE (PubMed), Embase, Scopus, the Cochrane Central Register of Controlled Trials, and Web of Science Core Collection from 2009 to present. All randomized controlled clinical trials and prospective non-randomized controlled trials which report on comparison of NIR-ICG versus IOC will be included. All patients over 18 years old who require elective or urgent minimally invasive cholecystectomy (undergoing NIR-ICG during this procedure) due to gallstone disease both acute and chronic will be included. Since BDI has a low incidence, the primary outcome will be the ability to visualize extrahepatic biliary anatomy and the time to obtain relevant images of these structures.

Two researchers will individually screen the identified records, according to a list of inclusion and exclusion criteria. Bias of the studies will be evaluated with the Newcastle-Ottawa score for non-randomized studies and with The Cochrane Risk of Bias Tool for randomized controlled trials. Quality of evidence for all outcomes will be determined with the GRADE system. The data will be registered in a predesigned database. If selected studies are sufficiently homogeneous, we will perform a meta-analysis of reported results. In the event of a substantial heterogeneity, a narrative synthesis will be provided. Subgroup analysis will be used to investigate possible sources of heterogeneity.

**Discussion:**

Understanding the benefits of this technique is critical to ensuring policymakers can make informed decisions as to where preventive efforts should be focused regarding specific imaging techniques. If ICG is proven to be faster and non-invasive, its routine use could be encouraged.

**Systematic review registration:**

PROSPERO CRD42020177991.

**Supplementary Information:**

The online version contains supplementary material available at 10.1186/s13643-022-01907-6.

## Background

### Description of the condition

Gallstones are one of the most common diseases treated by general surgeons. Therefore, after hernia surgery, laparoscopic cholecystectomy (LC) is the most common surgical procedure performed in Europe [1]. Since the introduction of laparoscopy, LC has become the standard surgical approach in the treatment of cholelithiasis.

Despite the fact that there are several advantages when compared to the open approach [2, 3], the rate of bile duct injury (BDI) during LC increased significantly mainly during the initial period of its implementation as a routine procedure. Some authors reported BDI rates as high as 0.4%, which is significantly higher than with open cholecystectomy (0.1%) [4, 5]. Modern, registry-based, large population series report rates of BDI ranging from 0.08 to 0.3% [6, 7]. Nevertheless, this low incidence has to be weighed against the high numbers of cholecystectomies [8, 9] and with the potentially severe repercussions of BDI.

BDI might require multiple and multidisciplinary interventions to restore the biliary tree, from conservative treatment, minimally invasive endoscopic procedures up to increasingly life-threatening conditions, such as liver transplant. This avoidable situation would have enormous repercussions on the patient’s quality of life, with direct and indirect costs, as well as for the public institutions, absorbing enormous healthcare expenses [10]. Moreover, iatrogenic BDIs represents one of the leading reasons for malpractice suits against surgeons, with high average settlements per injury [11, 12].

All these considerations might justify and enforce the routine use of all available means to reduce the risk of BDI. It is well known that LC are performed by all types of general surgeons, not specifically hepatobiliary surgeons. Furthermore, this procedure is carried out in almost any type of centre, academic or not academic, therefore volume and expertise may vary. Strategies to avoid BDI should accomplish some characteristics to be applicable: cost-effective, short learning curve, and easy to implement and reproduce.

Since the main aetiology of BDI seems to be the misinterpretation of the biliary anatomy, various methods have been described to better identify those structures intraoperatively. This might be even more important in the presence of biliary tree inflammation and unapparent/undetected bile duct anatomical variants.

Diverse surgical techniques and different imaging modalities have been described to provide enhanced views of the biliary anatomy and potentially prevent or early detect BDI [13]. With regards to surgical techniques, critical view of safety (CVS) was described by Strasberg in 1995 [4] and has been widely acclaimed to reduce the BDI rate during LC. This has not yet been formally shown, most likely, because it has not been adopted universally and above all, is often poorly performed [14, 15]. Other techniques such as fundus first approach or laparoscopic subtotal cholecystectomy have both reported dropping the incidence and conversion rate of BDI [16]. By contrast, various imaging modalities have been described such as intraoperative ultrasound, X-ray intraoperative cholangiography (IOC), and near infrared indocyanine green fluorescent cholangiography (NIR-ICG). Among those, standard IOC is the imaging modality that has been more widely investigated and reported. IOC was originally described by Mirizzi [17] and has been used to draw the biliary anatomy, achieve early recognition, and decrease the severity of injury [17]. IOC has also been used to identify and intraoperatively manage common bile duct stones. There is no definitive evidence to support the routine use of IOC to prevent BDI [18]. In fact, it is currently performed selectively or rarely in most centres, according to the surgeon’s preference [12]. However, in the increasing trend of malpractice litigation worldwide, IOC is deemed the most effective argument of defensive medicine [9]. Nonetheless, radiation exposure, higher costs, requirement of performing an incision in the cystic duct (CD) potentially increasing the incidence of BDI and prolonged operative times are some disadvantages that lead to IOC being discontinued as a routine imaging modality [19]. One of the reasons why the risk of BDI may not have been eliminated could be due to the lack of an objective method of positive recognition of the biliary anatomy before any dissection takes place. A non-invasive and easy to interpret imaging modality may, however, replace the use of X-rays for cholangiography when indicated and solve the longer learning curve and the higher interobserver variability inherent to ultrasound.

### Description of the intervention

The Society of American Gastrointestinal and Endoscopic Surgeons (SAGES) was the first to publish guidelines for the clinical application of laparoscopic cholecystectomy in May 1990. These guidelines have periodically been updated. The version published in January 2010 [20] stated that:

Regarding BDI the guidelines state that:

- Factors which have been associated with BDI include surgeon experience, patient age, male gender and acute cholecystitis. (Level II, Grade C.)
- The safety of LC requires correct identification of relevant anatomy. (Level I, Grade A.)
- Intraoperative cholangiogram may reduce the rate or severity of injury and improve injury recognition. (Level II, Grade B.)

Identification of biliary structures during LC in order to avoid BDI can be achieved using various techniques, as mentioned in the last edition of the WSES Guidelines for the detection and management of BDI during cholecystectomy [21]. However, this version considers ICG as a promising not yet established technique that is recommended to use only in selected cases.

Because current guidelines may underestimate the role of ICG, a review of current evidence on the topic is needed in order to be able to give stronger recommendations for future guidelines regarding its utility, superiority compared to IOC and if its use should be not selective but routine.

Ishizawa and colleagues were the first to describe intraoperative NIR-ICG in 2009 to provide a biliary roadmap [22]. NIR-ICG was designed as a promising intraoperative imaging modality, which highlights the biliary ducts using fluorescence. Indocyanine green (ICG) is a fluorescence dye that has been approved for multiple clinical uses by the Food and Drug Organization (FDA) since 1956. ICG is hydrophilic and binds to albumin in plasma as well as to alpha-1 lipoprotein, has a half-life of 2 h [23], is exclusively eliminated in the liver, and has no metabolism. It is used in medicine to evaluate cardiac output, pulmonary and hepatic function, and for the detection of vitality in free flap reconstructions [24]. Near-infrared cholangiography (NIR-C) is based on the systemic injection of a bile-excreted fluorophore such as ICG. Peak concentration in the bile occurs between 30 min and 2 h after injection, whereas peak concentration in the arterial system is reached within 1–2 min [25]. The reported ICG dose varies significantly, ranging from 0.05 to 0.5 mg/kg or administration from 2.5 to 20 mg in a single intravenous dose [26], regardless of patient weight. Zarrinpar et al. reported that the optimal ICG dose leading to the best duct-to-liver signal ratio was 0.25 mg/kg, administered at least 45 min before imaging [27]. There is also a wide range regarding time of injection, ranging from 24 h prior to the operation to immediately after induction of anaesthesia [27, 28]. Both intravenous administration, as well as direct gallbladder injection, have been described [29].

When excited with a near-infrared laser, ICG emits light at a peak wavelength of approximately 800 nm [30], which is displayed on the monitor for real-time interpretation. The fluorescence imaging system consists of a light source and a filter that emits infrared and xenon light. This system is incorporated into the charge-coupled device camera and scope that can filter out light with wavelengths below 830 nm [31]. The light of the laparoscope can be easily changed to the infrared view using a pedal or flick a toggle switch on the endoscope. During the procedure, alternate exposure from xenon and infrared light can be used to inspect the operative field before and during Calot’s triangle dissection. The fluorescence imaging mode can be used at the discretion of the surgeon to help obtain a critical view of safety before transecting the dissected structures.

NIR-ICG is associated with minimal risk of complications, although anaphylactic reaction to ICG has been reported to occur at an incidence of 3/1000 (0.003%), especially at doses higher than 0.5 mg/kg [32]. As ICG is excreted by hepatocytes unaltered into the bile, excretion and detection with the near-infrared camera might be compromised in patients with cirrhosis, non-alcoholic steatohepatitis (NASH), or fatty liver disease [33].

### How the intervention might work

Both IOC and NIR-ICG have been described as safe and feasible techniques to assess biliary anatomy and to avoid BDI. However, in theory, NIR-ICG has all the advantages and none of the drawbacks of IOC [31].

First, NIR-ICG is an incisionless technique, it does not require cannulation of the CD, avoiding the risk of CD avulsion and BDI. In addition, since it does not depend on any dissection in order to cannulate the CD, it provides early imaging before starting the dissection, achieving a high rate of structure identification and being able to guide this dissection.

Second, early (prior to dissection) identification rates with NIR-ICG are adequate and it can be used multiple times during dissection without increasing radiation or contrast load to the patient compared to IOC. This makes it an attractive alternative for pregnant and young patients as well as in order to avoid repeated exposure for the staff members.

Third, it has the advantage of being real-time, allowing direct dissection and easy switching between xenon and infrared light modes. This can be conducted during and after Calot’s triangle dissection, being able to evaluate biliary anatomy in addition to its blood supply [34, 35]. On the contrary, IOC can only be performed once the cannula is placed after dissection and X-ray exposure has to be minimized, so no dissection is performed during this exploration. Moreover, IOC is believed to require longer operative time than NIR-ICG, thus increasing surgical time.

Fourth, NIR-ICG is low-cost, neither disposable equipment is required nor the presence of a specialist technician. In fact, the equipment and training required for radioprotection increases the cost of this procedure since IOC requires the fluoroscope to be handled by trained personnel which makes perioperative logistics more difficult.

Finally, NIR-ICG is easy to perform and to learn. A short learning curve has been described and it has been shown to be a good learning tool for surgeons in training [36–38].

### Why is it important to do this review?

When compared to IOC, NIR-ICG is supposed to provide equal visualization of the bile ducts before dissection. NIR-ICG has the potential to replace IOC for biliary mapping since IOC comes with higher costs, more difficult perioperative logistics, greater radiation exposure, greater use of radiographic contrast fluids, frequent technical failure when CD is obliterated and risk of BDI due to cannulation of the CD. NIR-ICG might be the best option for visualization of the biliary tract, although further research is necessary to confirm this recommendation.

NIR-ICG has been shown to be useful in identifying biliary structures in LC, however, its role in bile duct detection and prevention has not been established. The results reported from prior studies have mostly concluded that NIR-ICG improves visualization of biliary structures; however, these studies included a relatively small sample size, ranging between 23 and 120 patients [37, 39]. Several groups [40–42] have found NIR-ICG to be feasible, although it has not been validated against IOC, which is the standard for visualization of the critical junction. Studies comparing both techniques have not shown definitive conclusions regarding prevention of BDI and to date, based on the available data, the topic remains controversial. Given the low frequency of BDI, a randomized trial comparing BDI rates for routine and selective IOC versus NIR-ICG would be difficult to carry out since the number of patients required to demonstrate a potential BDI reduction would be prohibitively high. According to Livingston et al. 527 IOCs might be required to prevent a single BDI [43]. Therefore, it has been estimated that more than 30,000 patients would be needed in each group to detect a clinically significant reduction [44]. Consequently, the outcome of visualization of anatomic structures is a useful surrogate marker of clinical efficacy and a systematic review would be the preferred study type to prove these differences.

There have been no systematic reviews including randomized controlled trials or Cochrane systematic reviews on this topic. Existing reviews highlight the uncertainty of which is the preferred method for the visualization of biliary structures during LC [13, 26, 45]. A systematic review and metanalysis published in 2017 showed that there seems to be moderate to low quality evidence that visualization of the CD, common bile duct (CBD) and common hepatic duct (CHD) with NIR-ICG is better than using IOC although no statistical differences were found [46]. This review will capture additional data, including papers published since 2017, to allow greater understanding of trends and update the current literature since a considerable number of publications and great technological improvement has occurred in the last years.

Understanding the benefits of this technique is critical to ensuring policymakers can make informed decisions as to where preventive efforts should be focused regarding specific imaging techniques. Solid evidence is required regarding the use of NIR-ICG in order to implement it as a standard technique and include it in the curriculum of future generations of surgeons. A review in this topic is also particularly important because this technique seems safe and feasible although has not been widely implemented yet. A survey performed to surgeons who attended a conference on fluorescence-guided surgery showed that only 23% had experience in NIR-C procedures despite the selection bias. The congress attendees were surgeons who personally enrolled to attend a conference on fluorescence-guided surgery, who might represent a population with more access and experience with fluorescence-guided systems for laparoscopic cholecystectomies thus overestimating the percentage [47].

Finally, the use of IOC could be considered as a quality criterion during cholecystectomy [48]. Nevertheless, it is performed less often than what is stated in the literature, most likely because day-case cholecystectomies only require a short operative time and may enable the patient to be discharged home in the afternoon. If a faster and non-invasive technique was available, routine use could be encouraged and wide implementation could be a reality.

## Objectives

The aim of this systematic review will be to evaluate if NIR-ICG can visualize extrahepatic biliary anatomy more efficiently and safely than intraoperative cholangiography in minimally invasive cholecystectomy for gallstone disease. To this end, the proposed systematic review will aim to answer the following questions:

1. What are the bile duct injury rates during minimally invasive cholecystectomy for gallstone disease when standard IOC is performed compared to NIR-ICG?
2. When comparing NIR-ICG to standard IOC, what are the detection rates of biliary structures, such as CD, CBD, CHD, and CD-CHD junction, in order to perform a safe dissection of Calot’s triangle and to avoid bile duct injury?
3. Is there a definitive advantage in terms of operative time saving when NIR-ICG is used to identify biliary structures in patients with gallstone disease both in urgent and elective setting compared to IOC?
4. Is NIR-ICG a safer technique due to the lack of cystic duct dissection and radiation compared to IOC?

## Methods

This review protocol has been registered within the PROSPERO database (registration number: CRD42020177991) and is being reported in accordance with the reporting guidance provided in the Preferred Reporting Items for Systematic Reviews and Meta-Analyses Protocols (PRISMA-P) statement [49] (see checklists in Additional file 1).

### Eligibility criteria

Studies will be selected according to the following criteria: population, intervention, comparison and outcomes.

#### Study designs

The included studies will be restricted to human studies. All randomized controlled clinical trials (RCT) and prospective non-randomized controlled trials which report on comparison of NIR-ICG versus IOC will be considered for inclusion. Conference abstracts with sufficient data available will be included (if became full article afterwards only full article will be included to prevent duplication of data). RCT and prospective non-randomized controlled trials comparing other techniques than NIR-ICG or IOC, as well as single-arm prospective trials will be excluded. Retrospective series and case reports will neither be considered for inclusion.

#### Participants

We will include male or female patients over the age of 18 years old who require elective or urgent minimally invasive cholecystectomy (undergoing NIR-ICG during this procedure) due to gallstone disease both acute and chronic. Patients under 18 years old or undergoing procedures using NIR-ICG different than minimally invasive cholecystectomy will be excluded.

#### Intervention

Of interest are interventions addressing NIR-ICG performed during minimally invasive cholecystectomy with no restriction of dose, time of injection or way of administration, intravenous, or directly into the gallbladder. The scope of the review, therefore, warrants an examination of the capacity of visualization of biliary structures before, during and after dissection for NIR-ICG, classified as valid if one or more of the following structures, CD, CHD, CBD, or CD-CHD junction could be identified. Time will be measured from the first time the xenon light is used until biliary structures considered to have been identified.

#### Comparators

Given that IOC is the gold standard intervention to visualize biliary structures, we will include studies comparing this technique to NIR-ICG. In order to perform IOC, the CD will be dissected in a standardized manner and with the aid of a grasper forceps it will be cannulated with a plastic catheter, then a radiopaque contrast media will be introduced through the above-mentioned catheter [50]. Time will be measured from the application of the grasper until its removal, after obtaining a satisfactory cholangiogram, which will be defined by the ability to identify the CD and/or other biliary structures.

#### Outcomes

Primary and secondary outcomes, as detailed bellow, are of primary interest. If reported on, these will be analysed and graded. If a given clinical outcome is not reported on, we will analyze their possible surrogate outcomes. As some outcomes may be reported as a composite measure, we will extract all composite and individual outcomes as reported in the studies (e.g., visualization of each one of biliary ducts vs visualization of all of them).

##### Primary outcomes

- Ability to visualize extrahepatic biliary anatomy, consisting of visualization of CD, CBD, CHD, and CD-CHD junction.
- Time to obtain relevant images of the biliary tree.

##### Secondary outcomes

- Intraoperative identification of BDI and rate of BDI induced by each technique.
- Adverse events secondary to ICG infusion.
- Adverse events secondary to radiation as well as iatrogenic lesions secondary to CD cannulation.
- Identification of aberrant biliary anatomy.

##### Timing

There will be no restrictions by timing.

##### Setting

There will be no restrictions by type of setting.

##### Language and geography

There will be no restrictions by language or geography.

### Information sources

Literature search strategies will be developed using medical subject headings (MeSH) and text words related to the use of NIR-ICG and IOC in minimally invasive cholecystectomies. The search terms that will be used are indocyanine green, ICG, fluorescence, cholecystectomy, and bile duct injury.

We will search the following electronic databases (from 2009 to present.): MEDLINE (via PubMed), EMBASE, SCOPUS, the Cochrane Central Register of Controlled Trials (CENTRAL), and the Web of Science Core Collection. The electronic database search will be supplemented by searching for trial protocols through Clinical Trials (http://www.clincialtrials.gov). The literature search will have no language or time restrictions and will include all available literature up to the search date, although limited to human subjects. To ensure literature saturation, we will scan the reference lists of included studies or relevant reviews identified through the search.

### Search strategy

The specific search strategies will be created by a Health Sciences Librarian with expertise in systematic review searching. The MEDLINE strategy will be developed with input from the project team. A draft MEDLINE search strategy is included in Additional file 2. After the MEDLINE strategy is finalized, it will be adapted to the syntax and subject headings of the other databases. ClinicalTrials.gov will be also searched to retrieve ongoing or recently completed trials. PROSPERO will be searched for ongoing or recently completed systematic reviews. As relevant studies are identified, reviewers will check for additional relevant cited and citing articles. The search will be updated toward the end of the review, after being validated to ensure that the MEDLINE strategy retrieves a high proportion of eligible studies found through any means though indexed in MEDLINE.

### Study records

#### Data management

Literature search results will be uploaded to a Mendeley database. Search for duplicates will be performed by two researchers (MCP, MA) with the Mendeley search for duplicates tool as well as juxtaposing author names and titles by hand. When similar titles or author list are found only the most updated series will be included. If both conference abstract and full paper reporting on the same patients are found only the full paper will be assessed.

#### Selection process

After excluding duplicates, the review authors (MCP, MA) will independently screen the titles and abstracts yielded by the search against the inclusion criteria and code them as “retrieve” (eligible, potentially eligible, or unclear) or “do not retrieve.” Full reports for all titles that appear to meet the inclusion criteria (coded as retrieve) will be obtained and two review authors (MCP, MA) will then screen the full-text reports. They will identify studies for inclusion and record reasons to exclude the ineligible studies. Disagreement will be resolved through discussion. Neither of the review authors will be blind to the journal titles or to the study authors or institutions. The reference lists of all included studies will be hand-searched in order to identify other potentially relevant studies. Any areas of disagreement between the two primary researchers will be resolved through discussion and if necessary, a third researcher (RJ) will be involved. Duplicates will be identified and excluded. The selection process will be recorded in sufficient detail to complete a PRISMA flow diagram. The characteristics of the excluded studies will be displayed in a table.

#### Data collection process

A pre-defined data collection form was prepared with consensus and consultation with all the researchers. Once data was collected it was transferred to an excel-based data collection form for study characteristics and outcome data will be used. Two review authors (MCP, MA) will independently extract the following study characteristics and outcome data from included studies.

1. Participants: total number and number of patients in each group, mean age, age range, gender, severity of condition, diagnostic criteria, inclusion criteria, exclusion criteria.
2. Interventions: intervention, comparison, and any cointerventions.
3. Outcomes: primary and secondary outcomes specified and collected, time points reported, and all reported patient-important outcomes.
4. Study characteristics: study design, study methodology, first author’s name, year of publication, study design, total duration of study and run-in period, number of study centers and location, study setting, and withdrawals.
5. Notes: funding for trial, notable conflicts of interest of trial authors.

If an outcome is reported at two or more time points within the timeframe of the outcome—for example, 30-day and 90-day mortality are reported—we will use the data that are reported at the latest time point of the outcome. We will note in the “Characteristics of included studies” table if outcome data were reported in an unusable way. We will resolve disagreements by consensus. One review author (MCP) will copy across the data from the data collection form into the excel file and will double-check that the data are entered correctly by comparing the study reports with how the data are presented in the systematic review. A second review author (MA) will spot-check study characteristics for accuracy against the trial report.

### Assessment of risk of bias in included studies

To facilitate the assessment of possible risk of bias the Newcastle-Ottawa score (NOS) will be used to test the quality of non-randomized studies. This scale [51] employs a star scoring system for the quality assessment of studies. A total of 9 stars can be awarded to a study. A study can be awarded a maximum of 4 for selection, 3 for outcome and maximum of 2 stars for comparability. Studies will be categorized as low, moderate, and high quality depending on the number of stars; 0–3 corresponds to low quality, 4–6 to medium quality and 7–9 to high quality.

The version 2 of Cochrane tool for assessing risk of bias in randomized trials (RoB 2) will be used for randomized controlled trials. It will assess the risk of bias according to the following domains [52]: randomization process, deviations from intended interventions, missing outcome data, measurement of the outcome and selection of the reported result. Each study will be judged to be at either high, low risk, or raising some concerns for bias for each of the domains above. We will provide a quote from the study report and justification for our judgement in the “risk of bias” table. We will summarize the “Risk of bias” judgements across studies for each of the domains listed. These judgements will be made independently by two review authors. Disagreements will be resolved first by discussion and then by consulting a third author for arbitration. The review will be conducted according to this published protocol and report any deviations from it in the “Differences between protocol and review” section of the systematic review.

### Data synthesis

#### Measures of effect

Results will be presented in order by key question and, within key questions, in order of primary then secondary outcomes. Only studies for which risk of bias was either low or moderate are planned to be reported, therefore studies with high risk of bias will be omitted.

The primary outcome will be intraoperative identification of extrahepatic biliary anatomy. Since BDI has a low incidence, it will be measured as a surrogate marker, consisting of visualization specifically of CD, CBD, CHD, and/or CD-CHD junction. The number of BDI will be a secondary outcome. This will be given in number of patients and percentage, considering those detected either intraoperative or postoperative. Rate of visualization for the CD, CBD, CHD, and the CD-CHD junction will be given as proportions.

#### Unit of analysis issues

The unit of analysis will be individual participants with gallstone disease who undergo minimally invasive cholecystectomy and NIR-ICG, IOC or both are performed during the procedure. If we find any cross-over studies, we will include the data from both interventions before and after crossover. If we find any cluster-randomized studies unexpectedly, we will include the data in the analysis if results are adjusted for intra-cluster correlation. In multi-arm studies, the models account for the correlation between trial-specific treatment effects from the same trial in the context of network meta-analysis, which allows comparison of multiple treatments.

#### Data analysis

Data synthesis will occur in several stages. First, summary tables will be created to detail characteristics of each study included in the final review. Absolute differences in outcomes and mean differences between groups (NIR-ICG vs IOC) will be reported in tables, as well as standardized mean differences for outcomes that are reported in more than one way (e.g., visualization of CD, CBD, CHD, CD-CHD junction separately or all together).

Second, if sufficient and comparable data is available, a meta-analysis will be conducted for all outcomes that compare NIR-ICG vs IOC, using RevMan 5.4.1 [53]. Since clinical and protocol heterogeneity is expected, meta-analysis will be conducted using the random effects model. This model assumes the treatment effects follow a normal distribution, considering both with-in study and between-study variation.

Third, if a meta-analysis is not feasible for the aforementioned outcomes, we will provide a narrative description of the study results. For the outcomes not related to the comparison NIR-ICG vs IOC, we will also perform a narrative description. In our review, we will follow the Cochrane methods if a meta-analysis is deemed inappropriate, as described in the Cochrane Handbook [54]. In addition, we will adhere to a rigorous reporting methodology as described by the synthesis without meta-analysis (SWIM) guidelines [55]. These guidelines are a 9-item reporting checklist that contains the following items: grouping studies for synthesis, standardized metric used, synthesis method, criteria used to prioritize results, investigation of heterogeneity, certainty of evidence, data presentation methods, reporting results, and limitations of synthesis.

We will create a “Summary of Findings” table using the following items: ability to visualize extrahepatic biliary anatomy, time to obtain relevant images of the biliary tree, intraoperative identification of BDI and rate of BDI induced by each technique, adverse events secondary to ICG infusion and to radiation, iatrogenic lesions secondary to CD cannulation and identification of aberrant biliary anatomy. Continuous outcomes (feasibility of performing ICG and IOC, time to identification, rate of identification of the various biliary structures) will be expressed as standardized mean difference (SMD) with 95% confidence interval (CI). Dichotomous outcomes (presence of BDI, anatomical variants identified) will be expressed as relative risk (RR) with 95% CI.

#### Assessment of heterogeneity

Forrest plots will be used to visualize pooled estimates and extent of heterogeneity among studies. Heterogeneity will be assessed with the I^2^ test. I^2^ statistic ranges between 0 and 100% with values of 0 to 40%: might not be important; 30 to 60%: may represent moderate heterogeneity; 50 to 90%: may represent substantial heterogeneity; 75 to 100%: considerable heterogeneity respectively, to indicate low and considerable heterogeneity.

#### Additional analysis

Subgroup analysis will be used to investigate possible sources of heterogeneity, based on the following parameters: timing of the NIR-ICG administration, dose of NIR-ICG, and type of device camera used to capture fluorescence.

Sensitivity analysis will be performed to explain the source of heterogeneity:

1. Analysis of the material retrieved (full text vs abstract only, preliminary data vs final results, published vs unpublished material, study design)
2. Risk of bias (performing analysis by omitting studies evaluated as of high risk of bias).

#### Publication bias

If a sufficient number of articles are included, we will assess study bias (e.g., publication bias). Egger’s test will be used to test for asymmetry for continuous outcomes [56] and Peters test [57] will be used for binary data.

#### Confidence in cumulative evidence

The quality of evidence for all outcomes will be determined with the Grading of Recommendations Assessment, Development and Evaluation (GRADE) system [58]. Quality will be evaluated as high, moderate, low, or very low. The evaluation of quality will be independently performed by two of the authors (MCP and MA).

## Discussion

BDI is the most feared complication after cholecystectomy. BDI rates range from 0.08 to 0.3% [6, 7]. This low incidence has to be weighed against the high numbers of cholecystectomies [8, 9] and with the potentially severe repercussions of BDI. To date, there is sufficient data to conclude that NIR-ICG is a safe and feasible technique to assess biliary anatomy although current guidelines only mention IOC.

The main reason to choose IOC as the control for NIR-ICG is that it is a well-established maneuver and a valuable tool to achieve a correct visualization of the biliary tree. However, the use of IOC is slowly decreasing, mainly because of the increasing knowledge of the liver anatomy and the use of the so-called critical view. For this reason, the utility of NIR-ICG in the elective surgery will be hard to establish outside of prospective, randomized studies that consider the current protocols of laparoscopic cholecystectomy.

When compared to IOC, NIR-ICG is supposed to provide equal visualization of the bile ducts before dissection. NIR-ICG has the potential to replace IOC for biliary mapping since IOC comes with higher costs, more difficult perioperative logistics, greater radiation exposure, greater use of radiographic contrast fluids, frequent technical failure when CD is obliterated, and risk of BDI due to cannulation of the CD. NIR-ICG might be considered to be the best option for visualization of the biliary tract, although further research is necessary to confirm this recommendation.

The main limitation at study level will be the type of patients included in each publication. For patients with acute cholecystitis, the visualization of the biliary elements may be more difficult than in patients submitted to elective cholecystectomy. The inclusion of both type of patients in the main study may be an issue in the analysis of the results of each study. The main limitations at review level will be the difference between the protocols that each group have related to the moment and the dose of ICG administrated. Furthermore, the quality of the evaluation may depend on the quality of the technology used in the theatre. Thus, it could be expected that recent studies may be related to the use of newer ICG camaras with improved vision.

Understanding the benefits of this technique is critical to ensuring policymakers can make informed decisions as to where preventive efforts should be focused regarding specific imaging techniques. A review of current evidence on the topic is needed before the technique is included in future guidelines.

### Amendments

Any amendments made to this protocol when conducting the review will be outlined in PROSPERO and reported in the final manuscript.

### Dissemination plans

Results will be disseminated through conference presentations and publication in a peer-reviewed journal.

## Supplementary Information

**Additional file 1.** PRISMA-P checklist.

**Additional file 2.** Search strategy for electronic databases (May 7th 2020).

## Data Availability

Data sharing is not applicable to this article, as no datasets were generated or analyzed during the current study.

## Abbreviations

LC: Laparoscopic cholecystectomy
BDI: Bile duct injury
CVS: Critical view of safety
IOC: X-ray intraoperative cholangiography
NIR-ICG: Near infrared indocyanine green fluorescent
SAGES: Society of American Gastrointestinal and Endoscopic Surgeons
NIR-C: Near-infrared cholangiography
FDA: Food and drug organization
ICG: Indocyanine green
CD: Cystic duct
CBD: Common bile duct
CHD: Common hepatic duct
RCT: Randomized controlled clinical trials
CENTRAL: – Cochrane Central Register of Controlled Trials
NOS: Newcastle-Ottawa score
NASH: Non-alcoholic steatohepatitis
