# Supplementary material for: Near infrared indocyanine green fluorescent cholangiography versus intraoperative cholangiography to improve safety in laparoscopic cholecystectomy for gallstone disease—a systematic review protocol
Source: Syst Rev. 2022 Mar 3;11:36. doi: 10.1186/s13643-022-01907-6 (PMC8896300; doi:10.1186/s13643-022-01907-6)
Supplement: Supplementary file 2 — Additional file 2. Search strategy for electronic databases (May 7th 2020). [file 13643_2022_1907_MOESM2_ESM.docx]

ADDITIONAL FILE 2 –

SEARCH STRATEGY FOR ELECTRONIC DATABASES (May 7th 2020)

**Pubmed**

("Iatrogenic Disease"[Mesh] OR (("iatrogenic"[tw] OR "Hospital-Acquired"[tw]) AND (lesion*[tw] OR disease*[tw] OR condition*[tw] OR "Wounds and Injuries"[Mesh] OR Abdominal Injuries[Mesh] OR injur*[tw])) OR "Biliary Tract Surgical Procedures"[Mesh] OR "Biliary Tract"[Mesh] OR "Biliary Tract Diseases"[Mesh] OR "Cholecystectomy"[Mesh] OR "Bile Ducts"[Mesh] OR Bile duct*[tw] OR Biliar*[tw] OR hepatobiliar*[tw] OR gallbladder[tw] OR "gall bladder"[tw] OR gallstone*[tw] OR cystic duct*[tw] OR hepatic duct*[tw] OR cholecyst*[tw] OR cholelithiasis[tw] OR cholangitis[tw]) AND ("Cholangiography"[Mesh] OR cholangiogra*[tw] OR "Ultrasonography"[Mesh] OR ultrasound*[tw] OR ultrasonogra*[tw] OR "Spectroscopy, Near-Infrared"[Mesh] OR near-infrared[tw] OR nir[tw] OR nirs[tw] OR "Surgery, Computer-Assisted"[Mesh] OR image guided surger*[tw] OR computer-aided surger*[tw] OR computer assisted surger*[tw] OR robot*[tw] OR biliary map*[tw] OR ((real time[tw] OR realtime[tw] OR biliary[tw] OR optical[tw] OR intra-operative[tw] OR intraoperative[tw]) AND (imag*[tw] OR visual*[tw]))) AND ("Indocyanine Green"[Mesh] OR "Coloring Agents"[PA] OR indocyanine green[tw] OR wofaverdin[tw] OR vophaverdin[tw] OR icg[tw] OR "Fluorescent Dyes"[Mesh] OR "Fluorescent Dyes"[PA] OR fluorescen*[tw] OR fluorochrome*[tw] OR fluorogenic*[tw] OR CW800-CA [Supplementary Concept] OR cw800*[tw] OR irdye*[tw] OR fluorophore*[tw] OR fluorescein[tw])

Result → 799 references

**Embase**

('iatrogenic disease'/exp OR (('iatrogenic':ti,ab,kw OR 'hospital-acquired':ti,ab,kw) AND ('lesion*':ti,ab,kw OR 'disease*':ti,ab,kw OR 'condition*':ti,ab,kw OR 'injury'/exp OR 'abdominal injury'/exp OR 'injur*':ti,ab,kw)) OR 'biliary tract surgery'/exp OR 'hepatobiliary system'/exp OR 'biliary tract disease'/exp OR 'cholecystectomy'/exp OR 'bile duct'/exp OR 'bile duct*':ti,ab,kw OR 'biliar*':ti,ab,kw OR 'hepatobiliar*':ti,ab,kw OR 'gallbladder':ti,ab,kw OR 'gall bladder':ti,ab,kw OR 'gallstone*':ti,ab,kw OR 'cystic duct*':ti,ab,kw OR 'hepatic duct*':ti,ab,kw OR 'cholecyst*':ti,ab,kw OR 'cholelithiasis':ti,ab,kw OR 'cholangitis':ti,ab,kw) AND ('cholangiography'/exp OR 'cholangiogra*':ti,ab,kw OR 'echography'/exp OR 'ultrasound*':ti,ab,kw OR 'ultrasonogra*':ti,ab,kw OR 'near infrared spectroscopy'/exp OR 'near infrared':ti,ab,kw OR 'nir':ti,ab,kw OR 'nirs':ti,ab,kw OR 'computer assisted surgery'/exp OR 'image guided surger*':ti,ab,kw OR 'computer aided surger*':ti,ab,kw OR 'computer assisted surger*':ti,ab,kw OR 'robot*':ti,ab,kw OR 'biliary map*':ti,ab,kw OR (('real time':ti,ab,kw OR 'realtime':ti,ab,kw OR 'biliary':ti,ab,kw OR 'optical':ti,ab,kw OR 'intra-operative':ti,ab,kw OR 'intraoperative':ti,ab,kw) AND ('imag*':ti,ab,kw OR 'visual*':ti,ab,kw))) AND ('indocyanine green'/exp OR 'coloring agent'/exp OR 'indocyanine green':ti,ab,kw OR 'wofaverdin':ti,ab,kw OR 'vophaverdin':ti,ab,kw OR 'icg':ti,ab,kw OR 'fluorescent dye'/exp OR 'fluorescen*':ti,ab,kw OR 'fluorochrome*':ti,ab,kw OR 'fluorogenic*':ti,ab,kw OR 'cw800*':ti,ab,kw OR 'irdye*':ti,ab,kw OR 'fluorophore*':ti,ab,kw OR 'fluorescein':ti,ab,kw)

Results → 3275 references

**Scopus**

ALL(((("iatrogenic" OR "Hospital-Acquired") AND (lesion* OR disease* OR condition* OR injur*)) OR Bile duct* OR Biliar* OR hepatobiliar* OR gallbladder OR "gall bladder" OR gallstone* OR cystic duct* OR hepatic duct* OR cholecyst* OR cholelithiasis OR cholangitis) AND (cholangiogra* OR ultrasound* OR ultrasonogra* OR near-infrared OR nir OR nirs OR image guided surger* OR computer-aided surger* OR computer assisted surger* OR robot* OR biliary map* OR ((real time OR realtime OR biliary OR optical OR intra-operative OR intraoperative) AND (imag* OR visual*))) AND ( indocyanine green OR wofaverdin OR vophaverdin OR icg OR fluorescen* OR fluorochrome* OR fluorogenic* OR cw800* OR irdye* OR fluorophore* OR fluorescein) )

Results → 211 references

**Web of Science Core Collection**

TS=(((("iatrogenic" OR "Hospital-Acquired") AND (lesion* OR disease* OR condition* OR injur*)) OR Bile duct* OR Biliar* OR hepatobiliar* OR gallbladder OR "gall bladder" OR gallstone* OR cystic duct* OR hepatic duct* OR cholecyst* OR cholelithiasis OR cholangitis) AND (cholangiogra* OR ultrasound* OR ultrasonogra* OR near-infrared OR nir OR nirs OR image guided surger* OR computer-aided surger* OR computer assisted surger* OR robot* OR biliary map* OR ((real time OR realtime OR biliary OR optical OR intra-operative OR intraoperative) AND (imag* OR visual*))) AND ( indocyanine green OR wofaverdin OR vophaverdin OR icg OR fluorescen* OR fluorochrome* OR fluorogenic* OR cw800* OR irdye* OR fluorophore* OR fluorescein) )

Results → 1144 references

**Cochrane Library**

([mh "Iatrogenic Disease"] OR (("iatrogenic" OR "Hospital-Acquired") AND (lesion* OR disease* OR condition* OR [mh "Wounds and Injuries"] OR [mh "Abdominal Injuries"] OR injur*)) OR [mh "Biliary Tract Surgical Procedures"] OR [mh "Biliary Tract"] OR [mh "Biliary Tract Diseases"] OR [mh "Cholecystectomy"] OR [mh "Bile Ducts"] OR Bile duct* OR Biliar* OR hepatobiliar* OR gallbladder OR "gall bladder" OR gallstone* OR cystic duct* OR hepatic duct* OR cholecyst* OR cholelithiasis OR cholangitis) AND ([mh "Cholangiography"] OR cholangiogra* OR [mh "Ultrasonography"] OR ultrasound* OR ultrasonogra* OR [mh "Spectroscopy, Near-Infrared"] OR "near-infrared" OR nir OR nirs OR [mh "Surgery, Computer-Assisted"] OR "image guided surger*" OR "computer-aided surger*" OR "computer assisted surger*" OR robot* OR "biliary map*" OR (("real time" OR realtime OR biliary OR optical OR intra-operative OR intraoperative) AND (imag* OR visual*))) AND ([mh "Indocyanine Green"] OR "indocyanine green" OR wofaverdin OR vophaverdin OR icg OR [mh "Fluorescent Dyes"] OR fluorescen* OR fluorochrome* OR fluorogenic* OR cw800* OR irdye* OR fluorophore* OR fluorescein)

Results → 71 references
